# Repurposing antihypertensive drugs for the prevention of Alzheimer’s disease: a Mendelian randomization study

**DOI:** 10.1093/ije/dyz155

**Published:** 2019-07-23

**Authors:** Venexia M Walker, Patrick G Kehoe, Richard M Martin, Neil M Davies

**Affiliations:** 1 Medical Research Council Integrative Epidemiology Unit, University of Bristol, Bristol, UK; 2 Bristol Medical School: Population Health Sciences, University of Bristol, Bristol, UK; 3 Dementia Research Group, University of Bristol, Bristol, UK; 4 Bristol Medical School: Translational Health Sciences, University of Bristol, Bristol, UK

**Keywords:** Mendelian randomization, drug repurposing, Alzheimer’s disease, hypertension, antihypertensive drugs

## Abstract

**Background:**

Evidence concerning the potential repurposing of antihypertensives for Alzheimer’s disease prevention is inconclusive. We used Mendelian randomization, which can be more robust to confounding by indication and patient characteristics, to investigate the effects of lowering systolic blood pressure, via the protein targets of different antihypertensive drug classes, on Alzheimer’s disease.

**Methods:**

We used summary statistics from genome-wide association studies of systolic blood pressure and Alzheimer’s disease in a two-sample Mendelian randomization analysis. We identified single-nucleotide polymorphisms (SNPs) that mimic the action of antihypertensive protein targets and estimated the effect of lowering systolic blood pressure on Alzheimer’s disease in three ways: (i) combining the protein targets of antihypertensive drug classes, (ii) combining all protein targets and (iii) without consideration of the protein targets.

**Results:**

There was limited evidence that lowering systolic blood pressure, via the protein targets of antihypertensive drug classes, affected Alzheimer’s disease risk. For example, the protein targets of *calcium channel blockers* had an odds ratio (OR) per 10 mmHg lower systolic blood pressure of 1.53 [95% confidence interval (CI): 0.94 to 2.49; *p* = 0.09; SNPs = 17]. We also found limited evidence for an effect when combining all protein targets (OR per 10 mmHg lower systolic blood pressure: 1.14; 95% CI: 0.83 to 1.56; *p* = 0.41; SNPs = 59) and without consideration of the protein targets (OR per 10 mmHg lower systolic blood pressure: 1.04; 95% CI: 0.95 to 1.13; *p* = 0.45; SNPs = 153).

**Conclusions:**

Mendelian randomization suggests that lowering systolic blood pressure via the protein targets of antihypertensive drugs is unlikely to affect the risk of developing Alzheimer’s disease. Consequently, if specific antihypertensive drug classes do affect the risk of Alzheimer’s disease, they may not do so via systolic blood pressure.

**Key Messages**

This is the first study to use Mendelian randomization to estimate the effects of the protein targets of the 12 most common antihypertensive drug classes on Alzheimer’s disease.
Mendelian randomization suggests that lowering systolic blood pressure via the protein targets of antihypertensive drugs is unlikely to affect the risk of developing Alzheimer’s disease.
If specific antihypertensive drug classes do affect Alzheimer’s disease risk, they may not do so via systolic blood pressure.

## Introduction

Drug repurposing applies existing drugs to novel indications to identify potential treatments in a more rapid and cost-effective manner than traditional drug development. This approach is of interest for Alzheimer’s disease, as there are currently no preventative or disease-modifying therapies, despite investment in 1120 unique drug targets between 1995 and 2014.^1–3^ Antihypertensive drugs have previously been highlighted as priority repurposing candidates for Alzheimer’s disease prevention and several observational studies and a handful of trials have investigated this hypothesis.^2^^,^^4^ However, the evidence to date is inconclusive.^4^

Mendelian randomization has been proposed to predict drug repurposing opportunities and overcome some of the issues associated with conventional observational studies.^5^ Mendelian randomization is a form of instrumental variable analysis that uses germline genetic variation, assigned randomly at conception and akin to randomization in a randomized controlled trial, as an instrument for potentially modifiable exposures of interest.^6–8^ Without individual-level data, two-sample Mendelian randomization can be implemented using summary data on single-nucleotide polymorphisms (SNPs) from separate genome-wide association studies (GWASs) for the instrument–exposure (sample one) and instrument–outcome (sample two) associations.^9^ This approach has been used before to study the relationship between blood pressure and Alzheimer’s disease but it has not been used to estimate the effect of lowering systolic blood pressure, via the same mechanisms as the 12 most common antihypertensive drug classes, on Alzheimer’s disease.^10–12^

In this study, we use SNPs as instruments, selected to mimic the action of the protein targets of antihypertensive drug classes, in a two-sample Mendelian randomization analysis of systolic blood pressure on Alzheimer’s disease. Our rationale is to understand whether there are differences between specific antihypertensive drug classes on Alzheimer’s disease risk, which could inform the prioritization of repurposing candidates, and provide evidence at the drug class level that could be triangulated with that from other sources.^13^ Greater understanding of antihypertensives and their effect on Alzheimer’s disease may also highlight potentially relevant biological mechanisms for this disease. Some of these drugs, such as those acting through angiotensin receptor and calcium channel blocking mechanisms, have been suggested to have protective effects on Alzheimer’s disease that are independent of blood pressure lowering.^14–16^ As we used instruments that proxy lowering systolic blood pressure via specific protein targets, our estimates include all downstream effects of altering these targets that act through lowering systolic blood pressure so we can better understand what is, and is not, related to blood pressure lowering.^5^

## Methods

### Study design

We conducted a two-sample Mendelian randomization analysis using summary data on SNPs from GWAS. We identified SNPs to proxy the protein targets of antihypertensive drugs on the basis that they mimicked the action of that drug on the target. For example, angiotensin-converting enzyme inhibitors work by inhibiting the enzyme angiotensin-converting enzyme. We therefore selected SNPs in the angiotensin-converting enzyme gene to use as a genetic proxy for the protein targets of this drug class. Effect sizes for these SNPs were then extracted from a GWAS of systolic blood pressure to estimate the instrument–exposure association.^17^ The instrument–outcome association was estimated using the effect sizes for these same SNPs from a GWAS of Alzheimer’s disease.^18^ All data used were publicly available and mostly obtained from European ancestry populations [the exception being the Genotype-Tissue Expression (GTEx) project data—see below].

### Systolic blood pressure phenotype

The systolic blood pressure phenotype was defined using a GWAS of the UK Biobank cohort.^17^ UK Biobank consists of 503 317 Caucasian people from the UK, aged between 38 and 73 years.^19^^,^^20^ The GWAS was based on 317 754 of the participants, who were required to be of ‘White British genetic ancestry’. The GWAS model included sex and the first 10 principal components as covariates.^21^

### Alzheimer’s disease phenotype

The Alzheimer’s disease phenotype was defined using the International Genomics of Alzheimer's Project (IGAP) GWAS Stage 1 results. These data were from a European ancestry meta-analysis of 17 008 Alzheimer’s disease cases, which were a mixture of clinically and autopsy-confirmed, and 37 154 controls.^18^

### Instrument selection

We identified 12 antihypertensive drug classes in the British National Formulary.^22^ They were: *adrenergic neurone blocking drugs; alpha-adrenoceptor blockers*, *angiotensin-converting enzyme inhibitors*, *angiotensin-II receptor blockers*, *beta-adrenoceptor blockers*, *calcium channel blockers*, *centrally acting antihypertensive drugs*, *loop diuretics; potassium-sparing diuretics and aldosterone antagonists*, *renin inhibitors*, *thiazides and related diuretics*, and *vasodilator antihypertensives*. Using the drug substance information, we were able to identify pharmacologically active protein targets and the corresponding genes in the DrugBank database (https://www.drugbank.ca/; version 5.1.1).^23^ We then identified SNPs to instrument each protein target using the GTeX project data (Release V7; dbGaP Accession phs000424.v7.p2), which contains expression quantitative trait loci analyses of 48 tissues in 620 donors.^24^ The full GTEx dataset, which consists of 714 donors, is 65.8% male and 85.2% White. SNPs marked as the ‘best SNP’ for the gene (defined by GTEx as the variant with the smallest nominal *p*-value for a variant-gene pair) in any tissue were selected for analysis.

To validate the SNPs as instruments for the protein targets of the antihypertensive drugs, we estimated their effect on systolic blood pressure using two-sample Mendelian randomization. The SNP-expression association, extracted from GTEx as described above, was on the scale of a standard deviation change in ribonucleic acid (RNA)-expression levels for each additional effect allele. The SNP–systolic blood pressure association was extracted from the systolic blood pressure GWAS in UK Biobank and represented the standard-deviation change in systolic blood pressure for each additional effect allele. These associations were then used to estimate the effect of the protein target on systolic blood pressure (i.e. the standard deviation change in systolic blood pressure per standard deviation change in RNA-expression levels). SNPs with evidence of an effect on systolic blood pressure were retained for the main analysis. This instrument selection process is presented in Supplementary Figure 1, available as Supplementary data at *IJE* online.

### Statistical methods

We used two-sample Mendelian randomization to estimate the effect of lowering systolic blood pressure on Alzheimer’s disease in three ways. First, we estimated the effect of the protein targets of specific drug classes by combining their effects. This used the instruments defined in the previous section. Second, we estimated the effect of all protein targets of antihypertensive drugs as a whole on Alzheimer’s disease. Again, this used the instruments defined in the previous section. Finally, we estimated the overall effect of systolic blood pressure on Alzheimer’s disease by combining the effects of any genome-wide significant SNPs for systolic blood pressure.

When multiple SNPs were being used as an instrument, ‘clumping’ was performed to identify nearly independent SNPs using the linkage disequilibrium between them. SNPs absent in the outcome data were replaced by proxy SNPs in high linkage disequilibrium from the 1000 Genomes Project European data where possible.^25^^,^^26^ Proxies were required to have a minimum *R*-squared value of 0.8 and palindromic SNPs were permitted if their minor allele frequency was <0.3.

Prior to the analysis, data were harmonized to represent an increase in systolic blood pressure. Mendelian randomization was then performed using the inverse variance weighted method or, for single SNP instruments, the Wald ratio.^27–29^ Once complete, the Mendelian randomization results were transformed to be the odds ratio (OR) for Alzheimer’s disease per 10 mmHg lower systolic blood pressure to make the effect comparable to taking an antihypertensive, which on average reduces systolic blood pressure by 9 mmHg.^30^ All analyses used genome reference consortium human build 37 (GRCh37), assembly Hg19 and were performed in R using the package ‘TwoSampleMR’.^25^

### Sensitivity analyses

Mendelian randomization estimates may be subject to horizontal pleiotropy, whereby the SNP(s) chosen to proxy the exposure affect the outcome by a different mechanism to that intended.^31^ To estimate the extent of horizontal pleiotropy, we applied MR-Egger regression to all estimates based on 10 or more SNPs. The regression intercept for these analyses ‘can be interpreted as an estimate of the average pleiotropic effect across the genetic variants’.^32^ This can detect directional pleiotropy, which occurs when the biasing effects are not balanced around the null.

To examine heterogeneity within the drug classes, we also considered the effects of individual protein targets on Alzheimer’s disease. This analysis allowed us to ascertain whether certain targets were driving the combined effects we considered. Combined effects with very heterogeneous protein target results can be considered to have less reliable estimates than those where the protein targets were more homogeneous.

### Code availability

The analysis used R version 3.4.4.^33^ All coding files are available from GitHub (https://github.com/venexia/MR-antihypertensives-AD).

## Results

### Instrument selection

We identified a total of 73 unique protein targets of antihypertensive drugs (Supplementary Table 1, available as Supplementary data at *IJE* online). Among these targets, 68 had an effect in one or more GTEx tissues and 58 of those 68 provided evidence that the target affected systolic blood pressure (Supplementary Table 2, available as Supplementary data at *IJE* online). Supplementary Figure 2, available as Supplementary data at *IJE* online, summarizes the results of the Mendelian randomization analysis of expression on systolic blood pressure. A further six targets were excluded prior to the main analysis because neither the genetic instrument nor a suitable proxy was available in the outcome GWAS. Consequently, 52 unique protein targets were ultimately analysed (Supplementary Table 3, available as Supplementary data at *IJE* online).

### Drug class effects

**Figure 1:**
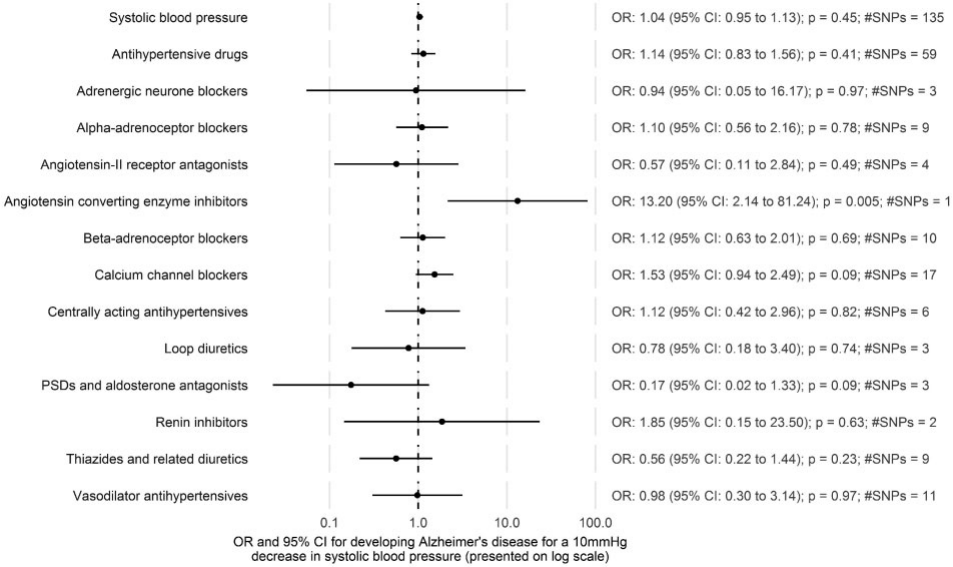
Estimates for the effect of systolic blood pressure on Alzheimer’s disease from two-sample Mendelian randomization.

There was limited evidence that reducing systolic blood pressure affected the risk of Alzheimer’s disease, via the protein targets, at the drug class level, with most estimates providing little evidence to exclude the null (Figure 1; Supplementary Table 4, available as Supplementary data at *IJE* online). For example, *calcium channel blockers* had an OR of 1.53 [95% confidence interval (CI): 0.94 to 2.49; *p* = 0.09; SNPs = 17] and *loop diuretics* an OR of 0.78 (95% CI: 0.18 to 3.40; *p* = 0.74; SNPs = 3) per 10 mmHg lower systolic blood pressure. The exceptions to this were *angiotensin-converting enzyme inhibitors* (OR per 10 mmHg lower systolic blood pressure: 13.20; 95% CI: 2.14 to 81.24; *p* = 0.005; rs4968783) and *potassium-sparing diuretics and aldosterone antagonists* (OR per 10 mmHg lower systolic blood pressure: 0.17; 95% CI: 0.02 to 1.33; *p* = 0.09; SNPs = 3).

### Antihypertensive drug effect

We found little evidence for an overall effect of lowering systolic blood pressure on Alzheimer’s disease, via the protein targets, when combining them all (OR per 10 mmHg lower systolic blood pressure: 1.14; 95% CI: 0.83 to 1.56; *p* = 0.41; SNPs = 59) (Figure 1; Supplementary Table 4, available as Supplementary data at *IJE* online).

### Systolic blood pressure effect

We also found little evidence for an overall effect of lowering systolic blood pressure on Alzheimer’s disease, without consideration of the associated protein targets, as indicated by the OR of 1.04 (95% CI: 0.95 to 1.13; *p* = 0.45; SNPs = 135) per 10 mmHg lower systolic blood pressure (Figure 1; Supplementary Table 4, available as Supplementary data at *IJE* online).

### Sensitivity analyses

The Egger intercepts were close to zero for almost all analyses where they could be calculated (Supplementary Table 5, available as Supplementary data at *IJE* online). In addition, the estimates from the inverse variance weighted and MR-Egger methods were similar for all analyses with both the point estimate and CI for the inverse variance weighted method almost contained within the CI for the MR-Egger method (Supplementary Figure 3, available as Supplementary data at *IJE* online).

The analysis of individual targets identified some targets that were likely to be driving the drug class effects (Supplementary Figure 4, available as Supplementary data at *IJE* online). For example, the target *NR3C2* is estimated to be extremely protective (OR per 10 mmHg lower systolic blood pressure: 2.01e-3; 95% CI: 5.22e-6 to 0.78; *p* = 0.04; rs71616586) and is therefore likely to have contributed to the extremely protective effect observed for *potassium-sparing diuretics and aldosterone antagonists* (OR per 10 mmHg lower systolic blood pressure: 0.17; 95% CI: 0.02 to 1.33; *p* = 0.09; SNPs = 3).

## Discussion

We found limited evidence to support an overall effect of lowering systolic blood pressure on Alzheimer’s disease risk when combining the effects of any genome-wide significant SNPs for this exposure (OR per 10 mmHg lower systolic blood pressure: 1.04; 95% CI: 0.95 to 1.13; *p* = 0.45; SNPs = 135). There was also limited evidence that lowering systolic blood pressure via the protein targets of specific antihypertensive drug classes affected Alzheimer’s disease. For example, *calcium channel blockers* had an OR of 1.53 (95% CI: 0.94 to 2.49; *p* = 0.09; SNPs = 17) and *vasodilator antihypertensives* had an OR of 0.98 (95% CI: 0.30 to 3.14; *p* = 0.97; SNPs = 11) per 10 mmHg lower systolic blood pressure. This was reflected in the overall effect of lowering systolic blood pressure on Alzheimer’s disease when combining all identified protein targets, which had an OR of 1.14 (95% CI: 0.83 to 1.56; *p* = 0.41; SNPs = 59) per 10 mmHg lower systolic blood pressure. Despite this, we also report some extreme results, such as that for the protein targets of *angiotensin-converting enzyme inhibitors*, which were associated with an increased Alzheimer’s disease risk (OR per 10 mmHg lower systolic blood pressure: 13.29; 95% CI: 2.14 to 81.24; *p* = 0.005; rs4968783). Note that, as with all studies that make multiple inferences simultaneously, multiple testing should be considered when interpreting the evidence presented here.

**Figure 2:**
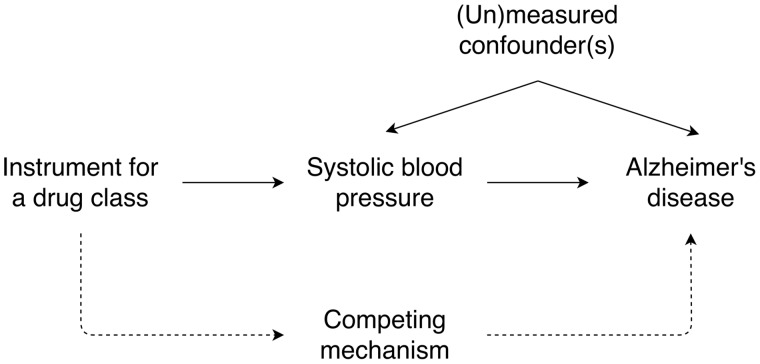
Mendelian randomization model in the presence of a competing mechanism.

A possible cause of these extreme results could be due to a competing mechanism, as illustrated in Figure 2. We estimated the effect of the protein targets of a given drug class on Alzheimer’s disease using the effect of the instrument for those targets on both systolic blood pressure (instrument–exposure association) and Alzheimer’s disease (instrument–outcome association). Our analysis assumed that the effect we were estimating acted through systolic blood pressure, although there is potentially a competing mechanism by which the protein targets can affect Alzheimer’s disease. If a competing mechanism does exist and the instrument–exposure association is small, estimates from Mendelian randomization can become inflated as the competing mechanism means the instrument–outcome association remains large. This is more apparent if you consider the Wald ratio used to calculate the effect for single SNP instruments:

$$\text{Exposure}–\text{outcome}\;\text{association}=\frac{\text{Instrument}–\text{outcome}\;\text{association}}{\text{Instrument}–\text{exposure}\;\text{association}}$$

In our analysis, we found a small effect of systolic blood pressure on Alzheimer’s disease and our extreme results were for the protein targets of drug classes that may well act through competing mechanisms. For instance, returning to the example of *angiotensin-converting enzyme inhibitors*, angiotensin-converting enzyme is proposed to affect both vascular pathways (such as blood pressure) and have independent effects on amyloid beta.^15^ In addition, *potassium-sparing diuretics and aldosterone antagonists*, the protein targets of which were also estimated to have an extreme effect (OR per 10 mmHg lower systolic blood pressure: 0.17; 95% CI: 0.02 to 1.33; *p* = 0.09; SNPs = 3), have previously been suggested to have a role, independently of blood pressure, in preventing cognitive decline.^34^ This explanation for the extreme results observed for the combined protein targets of certain drug classes, along with the limited evidence for an effect among the remaining drug classes, indicates that antihypertensive drug classes may not have an effect on Alzheimer’s disease via systolic blood pressure. However, it should be noted that, in both cases, we were unable to use MR-Egger as a sensitivity analysis, as the instruments contained fewer than 10 SNPs each. This means we could not confirm or falsify the role of pleiotropy in these findings. We also cannot rule out the possibility that these findings are the result of multiple testing.

### Comparison with existing literature

Two previous Mendelian randomization studies have studied the overall effect of systolic blood pressure on Alzheimer’s disease to date. These studies used different instruments and different systolic blood pressure GWAS, both to us and each other.^10^^,^^11^ This could lead to small differences in the results, as different SNPs are prioritized. The major difference between our choice of GWAS and those used for the existing studies is that the GWAS we used did not adjust for body mass index or correct for medication use. It is hypothesized that using adjusted and/or corrected GWAS for two-sample Mendelian randomization could lead to biased results if the other GWAS is not adjusted/and or corrected for the same factors, although further research is needed to confirm whether this is the case. The Østergaard *et al.* study found higher systolic blood pressure to be associated with a reduced risk of Alzheimer’s disease, whereas Larsson *et al.* found little evidence of an effect of systolic or diastolic blood pressure with Alzheimer’s disease. Our results agree with Larsson *et al.* in that there is unlikely to be an overall effect of systolic blood pressure on risk of Alzheimer’s disease. Gill *et al.* recently conducted a study that combined Mendelian randomization using genetic variants related to the protein targets of antihypertensive drugs with a PheWAS conducted in UK Biobank, although their analysis was restricted to *beta-adrenoceptor blockers* and *calcium channel blockers*.^12^ Our results broadly agree with those reported by Gill *et al.* for Alzheimer’s disease. There was a small overlap in the choice of SNPs used to instrument systolic blood pressure between our study and those previously reported, although there was less overlap when considering our instruments for the protein targets of specific drug classes (Supplementary Table 6, available as Supplementary data at *IJE* online). Using the previously reported instruments with our data, we were able to reproduce the previously reported results (Supplementary Figures 5 and 6, available as Supplementary data at *IJE* online).

Larsson *et al.* recently conducted a systematic review and meta-analysis, which identified five randomized controlled trials that have investigated whether antihypertensives prevent dementia (not Alzheimer’s disease specifically).^4^ Four of the five trials had point estimates that suggested a protective effect of antihypertensives compared with non-use, although results from three of these trials included the null within their CIs. This resulted in the meta-analysis finding an overall relative risk of 0.84 (95% CI: 0.69 to 1.02; *p* = 0.10). It is worth highlighting that most studies described in the meta-analysis were from populations with high cardiovascular morbidity and were designed around cardiovascular-related primary outcomes. In these trials, the proportion of dementia cases that derived from vascular mechanisms might be disproportionately high compared with other study populations.^35^^,^^36^ Since the publication of the meta-analysis, the first trial (NILVAD) to consider an antihypertensive drug (*calcium channel blocker* Nilvadipine) as a direct intervention in Alzheimer’s disease has been published—it found no benefit of the treatment among patients with mild to moderate probable Alzheimer's disease.^37^

The results of the SPRINT-MIND trial, which assessed the effect of intensive vs standard blood pressure control using a range of antihypertensive medications on probable dementia, mild cognitive impairment and a composite outcome combining probable dementia and mild cognitive impairment, have also been released.^38^ The trial found evidence to suggest that intensive blood pressure control was beneficial for the mild cognitive impairment and composite outcomes. Meanwhile, the estimate for the primary cognitive outcome of probable dementia included the null within its CI but may have been underpowered due to the early termination of the trial. There are several key differences between this trial and the analysis we present. First, our outcome of interest was Alzheimer’s disease and so cannot be directly compared against the mild cognitive impairment or dementia outcomes used in the trial. Second, the trial was designed to compare treatment goals, whereas our analysis was comparing treatment with no treatment. Depending on blood pressure at baseline, these might yield different results. Third, as noted for the trials included in the meta-analysis conducted by Larsson *et al.*, the primary outcome for the SPRINT trial was cardiovascular, meaning participants in SPRINT-MIND are more likely to have cognitive impairment and dementia outcomes derived from vascular mechanisms. Finally, Mendelian randomization estimates are of lifelong exposure, whereas this trial intervened on blood pressure for a median of 3.34 years in people with a mean age of 67.9 years. It is therefore possible that the trial has identified a critical period in which altering blood pressure has a beneficial impact on cognitive outcomes, which we cannot distinguish. Overall, while not directly comparable to our study, the findings from these recent trials, as well as the Larsson *et al.* meta-analysis, provide further evidence concerning the repurposing of antihypertensives for Alzheimer’s disease that can be considered together in a triangulation framework.

### Strengths and limitations

A strength of our study was the use of two-sample Mendelian randomization that meant we were able to utilize the IGAP GWAS for our outcome data, which contains information on 17 008 Alzheimer’s disease cases and 37 154 controls.^9^ The use of Mendelian randomization, over more conventional pharmacoepidemiological approaches, will have also addressed certain forms of confounding. This includes confounding by indication and confounding by the environmental and lifestyle factors of patients, which cannot be fully adjusted for using observational data. This is because measurement error and incomplete capture of all these potential confounding factors inevitably lead to residual confounding.

The limitations of this study included the risk of horizontal pleiotropy. We addressed this by conducting sensitivity analyses using MR-Egger when possible and by using the information concerning the existing indication of these drugs, i.e. alterations in systolic blood pressure, to inform our analysis. While the latter offers many benefits, it does restrict the inference we could make about the protein target effects that do not occur as a result of altering systolic blood pressure. Sensitivity analyses that considered the individual protein target effects also identified some heterogeneity that may have affected our combined estimates—e.g. the estimate for *potassium-sparing diuretics and aldosterone antagonist* may have seemed more protective due to the particularly large protective effect observed for one of the three targets under consideration: *NR3C2*. We were also limited by the fact that Mendelian randomization estimates the effect of lifelong exposure, whereas drugs typically have much shorter periods of exposure and systolic blood pressure may have age-dependent effects. This means that the effect sizes that we have estimated will not directly reflect what is observed in trials or clinical practice and may not be able to identify critical periods of exposure.^39^ The latter can be particularly problematic if a drug (or, in this case, a protein target of a drug) is beneficial at one point during the life course and harmful at another for instance. This is because Mendelian randomization will likely return a null finding for the lifelong effect (unless one of the effects is particularly pronounced) that cannot be easily distinguished from a null finding resulting from no effect across the life course.

Further limitations of this study relate to the populations used. For example, there is potentially ‘healthy volunteer’ bias in the UK Biobank study population, which would make the study unrepresentative of the UK population and may restrict the generalizability of our results.^20^ Our study is also potentially at risk of a survival bias effect whereby systolic blood pressure affects survival and survival is a prerequisite for Alzheimer’s disease. However, there is much uncertainty surrounding the relationship between systolic blood pressure and mortality, particularly in older populations.^40^ Consequently, we have chosen not to adjust for this in our model. Future studies could, however, consider inverse-probability weighting to correct for this potential survival bias. In addition to the above, the determination of cases for some studies in the IGAP data may have led to those with more extensive vascular disease history being misclassified as having vascular dementia. If this was the case, the effect of hypertension on Alzheimer’s disease could be misrepresented in our population and could have affected the results of this study. A final limitation of this study is the fact that the GTEx data are only 85.2% White, whereas the other data in our study are drawn from Caucasian populations. While this is a concern, we anticipate any effect on the analysis to be small, as these data are used for just one step in a much larger pipeline and the main analysis uses consistent populations.

## Conclusion

This study helps to inform the growing knowledge around repurposing antihypertensive drugs for Alzheimer’s disease prevention by using a different method, subject to different biases, to assess this research question. Combining the effects of all genome-wide significant SNPs for systolic blood pressure, we found little evidence to suggest that lowering systolic blood pressure itself will affect the risk of developing Alzheimer’s disease. This was accompanied by limited evidence for many of the protein targets of antihypertensive drug classes that we tested. This suggests that, if protein targets of specific antihypertensive drug classes do affect risk of Alzheimer’s disease, they may not do so via systolic blood pressure. Future research should consider this study, with other sources of evidence, in a triangulation framework to obtain a reliable answer concerning the potential repurposing of antihypertensives for Alzheimer’s disease prevention.

## Funding

This work was supported by the Perros Trust and the Integrative Epidemiology Unit. The Integrative Epidemiology Unit is supported by the Medical Research Council and the University of Bristol (grant number MC_UU_00011/1, MC_UU_00011/3). 

**Conflict of interest:** Walker is currently working on a manuscript in collaboration with GlaxoSmithKline plc that explores whether Mendelian randomization can predict drug success but does not receive financial support from the company. Davies has worked on unrelated projects funded as part of the Global Research Awards For Nicotine Dependence, which is an independent grant giving body funded by Pfizer. 

## Supplementary Material

dyz155_Supplementary_Data
