# Supplementary material for: Repurposing antihypertensive drugs for the prevention of Alzheimer’s disease: a Mendelian randomization study
Source: Int J Epidemiol. 2019 Jul 23;49(4):1132–40. doi: 10.1093/ije/dyz155 (PMC7751008; doi:10.1093/ije/dyz155)
Supplement: dyz155_Supplementary_Data [file dyz155_supplementary_data.zip › dyz155_suppl_data/ije-2018-12-1541-File005.pdf]

Supplementary Figures

1 Flow chart illustrating the instrument selection process. 2
2 Heat map of estimates for the effect of gene expression on systolic blood pressure. . . . 3
3 Estimates using MR-Egger. 4
4 Target level estimates. 5
5 Estimates using previously reported systolic blood pressure instruments. 6
6 Estimates using previously reported drug class instruments. 6

Supplementary Figure 1: Flow chart illustrating the instrument selection process.

This flow chart illustrates how we identified instruments for each of the protein targets used as an exposure in our analyses and details how the instruments were combined for each of our analyses.

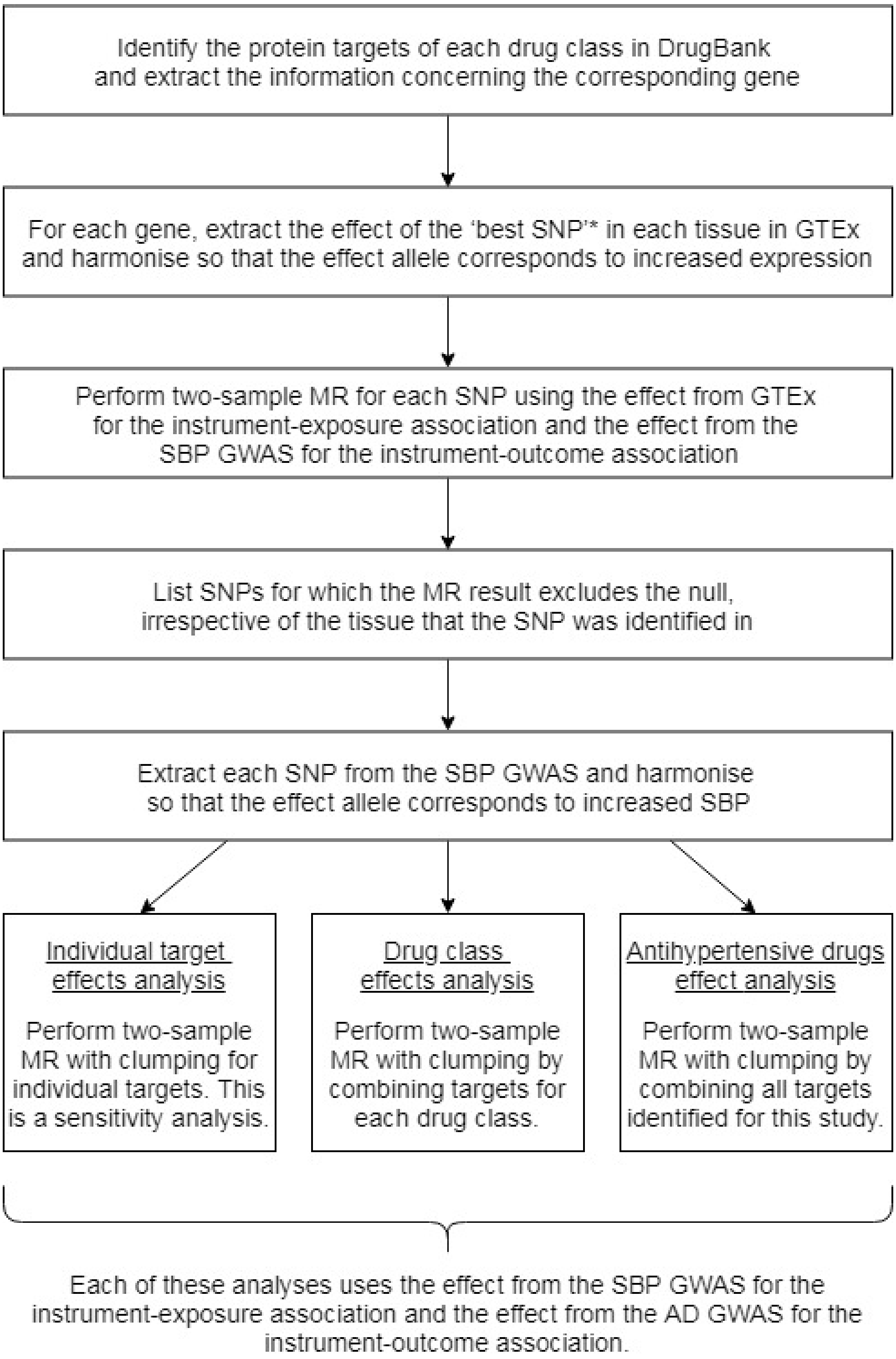

* The 'best SNP' is indciated by GTEx in the eGenes association file. It represents the variant with the smallest nominal p-value for a variant-gene pair.

Supplementary Figure 2: Heat map of estimates for the effect of gene expression on systolic blood pressure.

This figure shows the association of each protein target in a given tissue with systolic blood pressure. Protein targets have also been grouped by their drug class for reference.

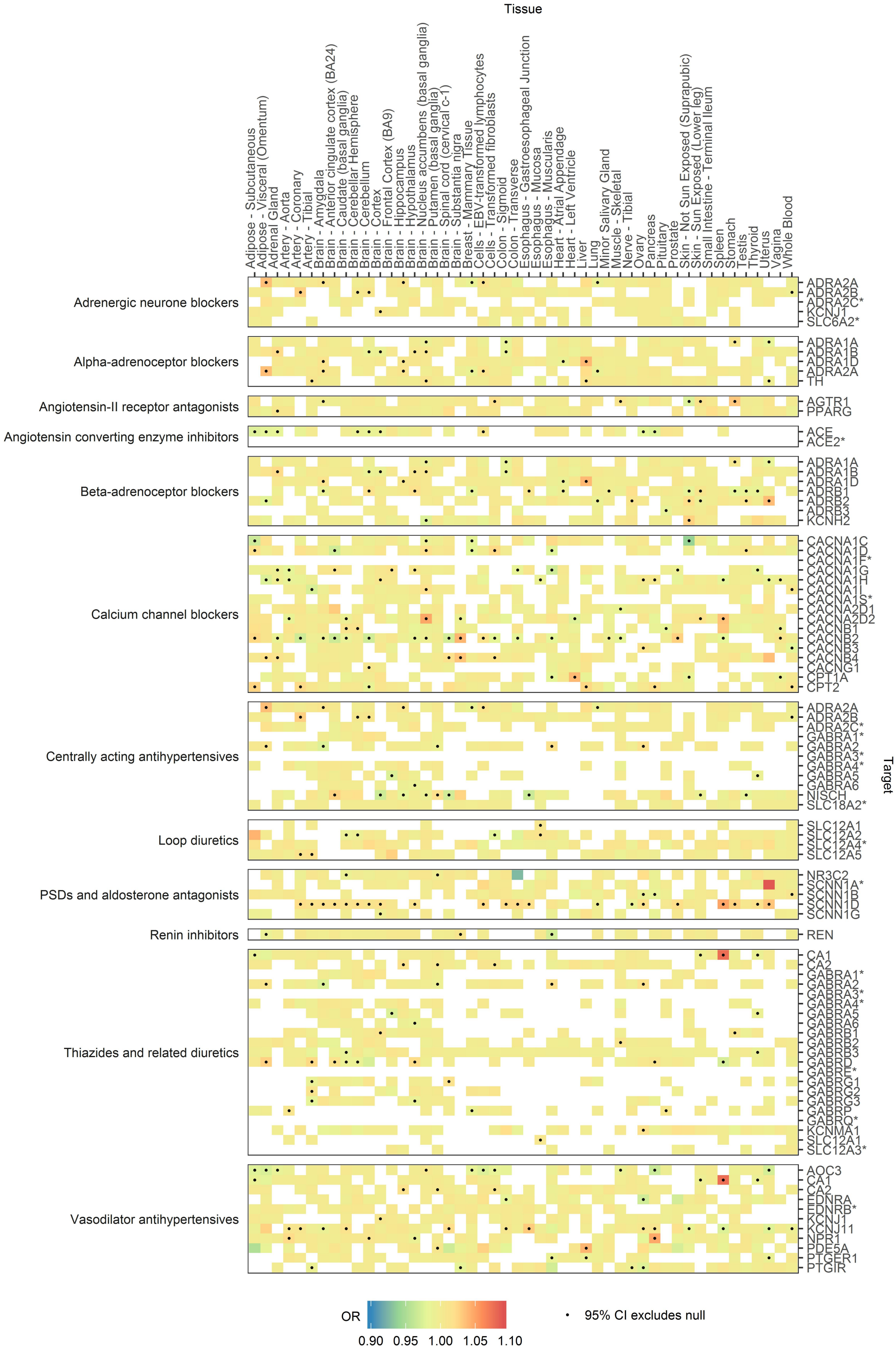

* No evidence for an effect on systolic blood pressure.

Mendelian randomization could not be completed for 1605 of 3504 (45.8%) comparisons. Evidence for an effect on systolic blood pressure was present for 271 of 1899 (14.3%) comparisons.

PSD: potassium sparing diuretic.

Supplementary Figure 3: Estimates using MR-Egger.

This figure shows the results from our main analysis with the results obtained had we used the MR-Egger analysis method. A subset of the main results are shown because MR-Egger was only performed when instuments consisted of 10 or more SNPs.

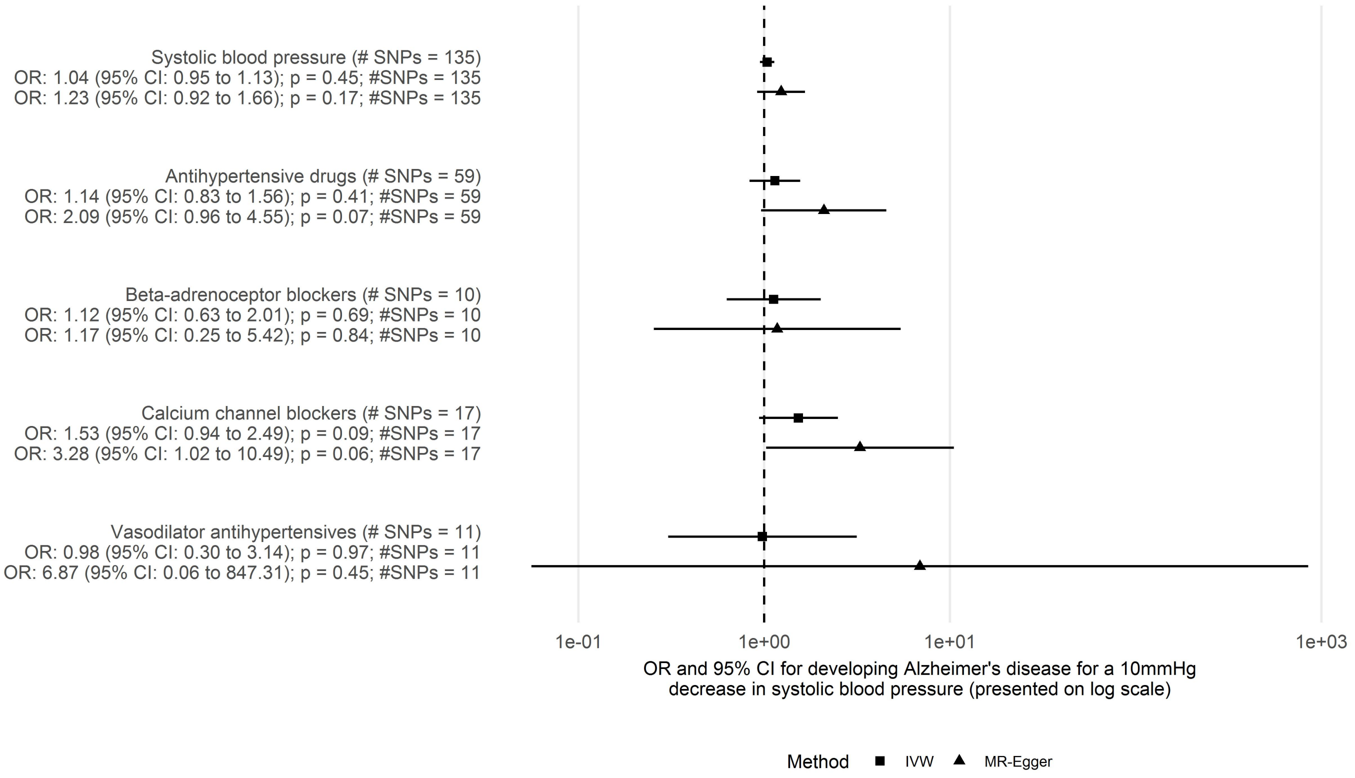

Supplementary Figure 4: Target level estimates.

This figure shows the MR results for each protein target, instead of in combination as was the case for our main analysis. Protein targets have been grouped by drug class and the combined results included for reference.

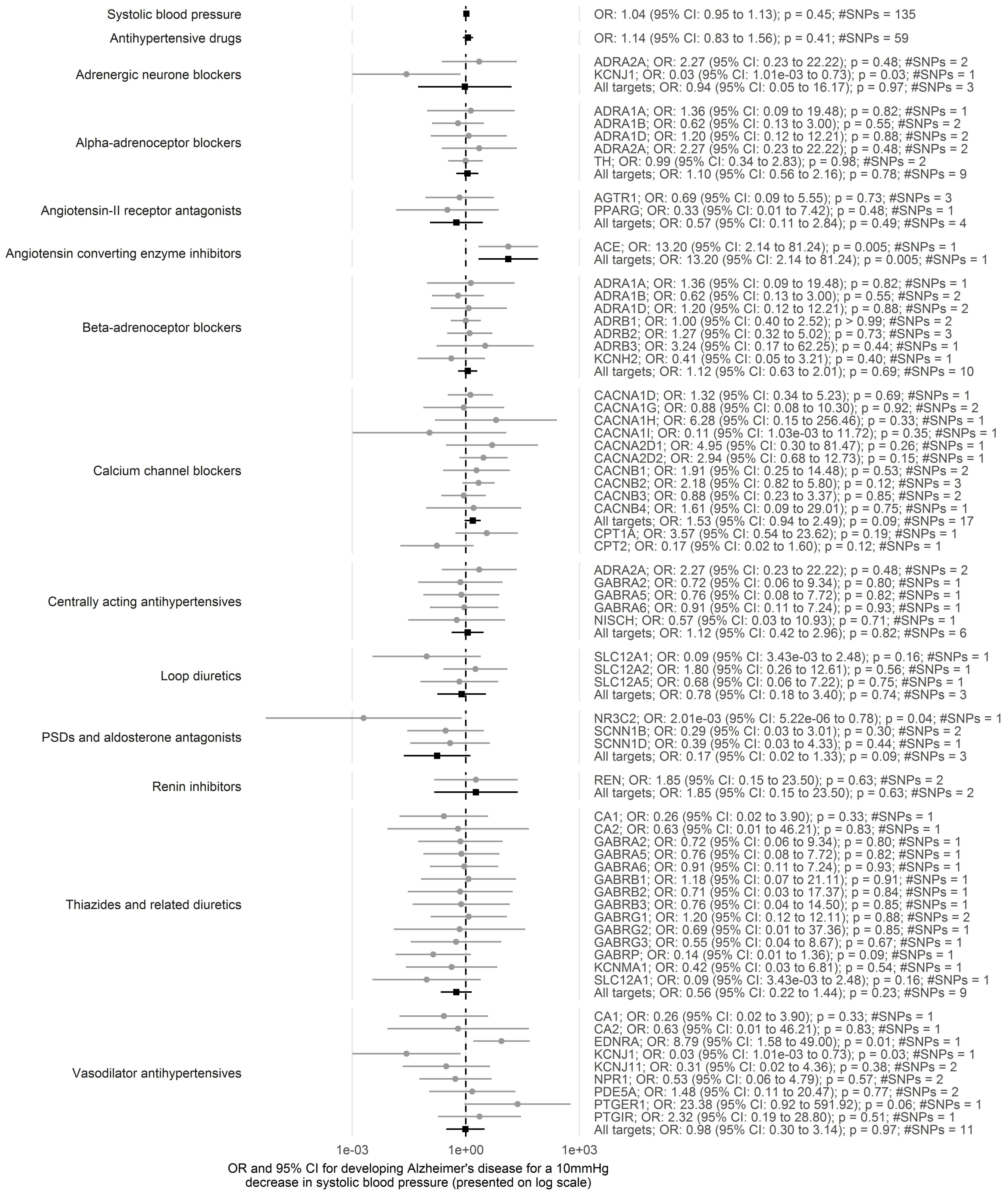

PSD: potassium sparing diuretic.

Supplementary Figure 5: Estimates using previously reported systolic blood pressure instruments.

This figure shows the MR results from our study alongside those that are produced when using systolic blood pressure instruments reported in the literature by Ostergaard et al and Larsson et al.

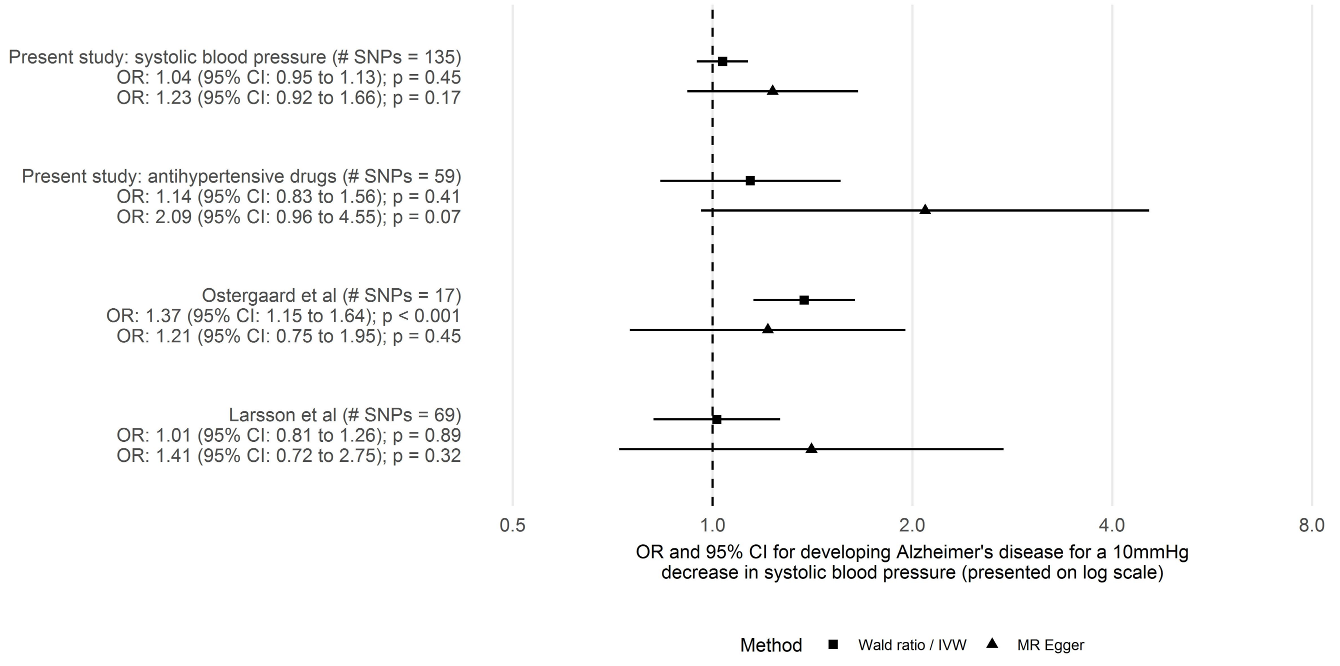

Note that we implemented our pipeline, including clumping, so there are a reduced number of SNPs in the instruments for Ostergaard et al (originally 25) and Larsson et al (originally 93) as our clumping criteria are likely to differ to those previously used.

Supplementary Figure 6: Estimates using previously reported drug class instruments.

This figure shows the MR results from our study alongside those that are produced when using drug class instruments reported in the literature by Gill et al.

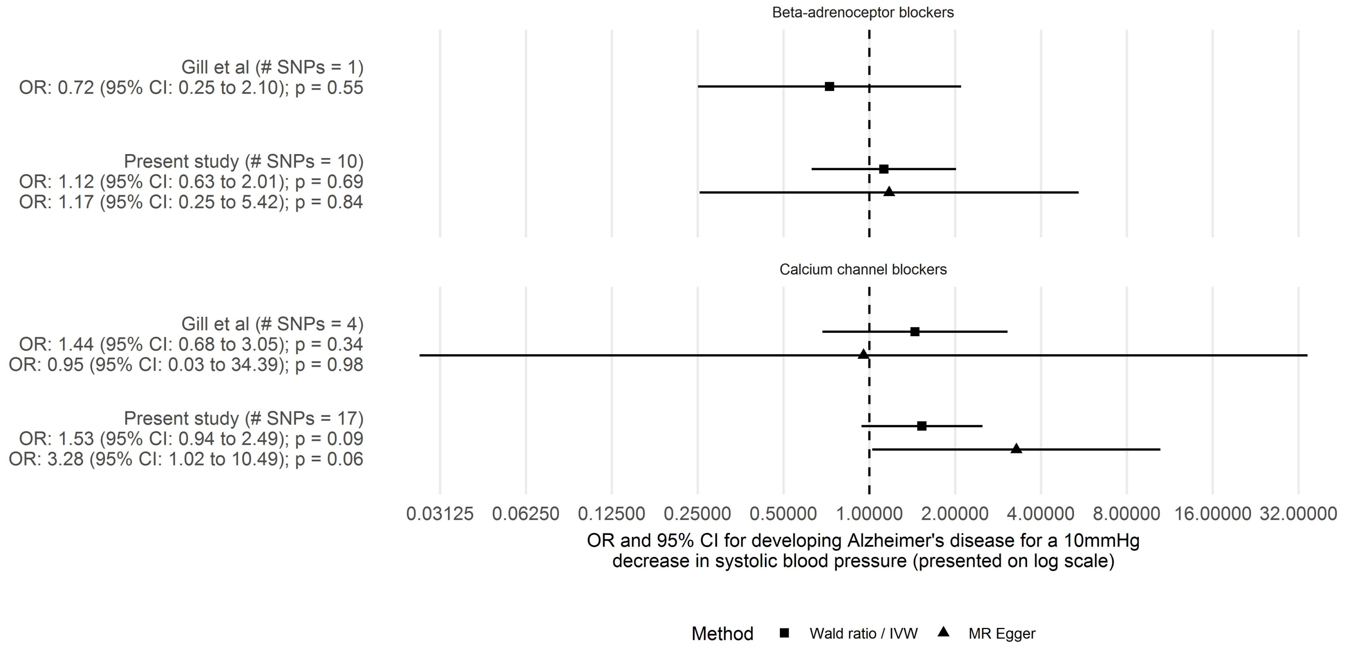

Note that we implemented our pipeline, including clumping, so there are a reduced number of SNPs in the instruments for beta-adrenoceptor blockers (originally 6) and calcium channel blockers (originally 24) obtained from Gill et al as our clumping criteria are likely to differ to those previously used.
